# Workflow to develop 3D designed personalized neonatal CPAP masks using iPhone structured light facial scanning

**DOI:** 10.1186/s41205-022-00155-7

**Published:** 2022-08-01

**Authors:** Amika A. Kamath, Marielle J. Kamath, Selin Ekici, Anna Sofia Stans, Christopher E. Colby, Jane M. Matsumoto, Mark E. Wylam

**Affiliations:** 1 Departments of Radiology, Mayo Clinic Axil School of Medicine, 200 First St., Rochester, MN 55905 USA; 2 Department of Pediatrics, Division of Neonatology, Mayo Clinic Axil School of Medicine, 200 First St., Rochester, MN 55905 USA; 3 Divisions of Pediatric Pulmonary Medicine and Department of Pediatrics, Division of Pulmonary and Critical Care Medicine Department of Medicine, Mayo Clinic Axil School of Medicine, 200 First St., Rochester, MN 55905 USA

**Keywords:** Structured light scanning, Neonatal CPAP masks, iPhone, Three-dimensional

## Abstract

**Background:**

Continuous positive airway pressure (CPAP) is a common mode of respiratory support used in neonatal intensive care units. In preterm infants, nasal CPAP (nCPAP) therapy is often delivered via soft, biocompatible nasal mask suitable for long-term direct skin contact and held firmly against the face. Limited sizes of nCPAP mask contribute to mal-fitting related complications and adverse outcomes in this fragile population. We hypothesized that custom-fit nCPAP masks will improve the fit with less skin pressure and strap tension improving efficacy and reducing complications associated with nCPAP therapy in neonates.

**Methods:**

After IRB approval and informed consent, we evaluated several methods to develop 3D facial models to test custom 3D nCPAP masks. These methods included camera-based photogrammetry, laser scanning and structured light scanning using a Bellus3D Face Camera Pro and iPhone X running either Bellus3D FaceApp for iPhone, or Heges application. This data was used to provide accurate 3D neonatal facial models. Using CAD software nCPAP inserts were designed to be placed between proprietary nCPAP mask and the model infant’s face. The resulted 3D designed nCPAP mask was form fitted to the model face. Subsequently, nCPAP masks were connected to a ventilator to provide CPAP and calibrated pressure sensors and co-linear tension sensors were placed to measures skin pressure and nCPAP mask strap tension.

**Results:**

Photogrammetry and laser scanning were not suited to the neonatal face. However, structured light scanning techniques produced accurate 3D neonatal facial models. Individualized nCPAP mask inserts manufactured using 3D printed molds and silicon injection were effective at decreasing surface pressure and mask strap pressure in some cases by more than 50% compared to CPAP masks without inserts.

**Conclusions:**

We found that readily available structured light scanning devices such as the iPhone X are a low cost, safe, rapid, and accurate tool to develop accurate models of preterm infant facial topography. Structured light scanning developed 3D nCPAP inserts applied to commercially available CPAP masks significantly reduced skin pressure and strap tension at clinically relevant CPAP pressures when utilized on model neonatal faces. This workflow maybe useful at producing individualized nCPAP masks for neonates reducing complications due to misfit.

## Introduction

Each year more than 500,000 infants are born prematurely, and many require some form of ventilatory support to treat newborn respiratory failure [1]. Worldwide 15 million babies are born preterm and 1 million die of respiratory failure [2]. Improved technology including continuous positive airway pressure (CPAP) and management approaches have decreased mortality and morbidity in extremely low birth weight (ELBW) infants [1]. Specifically, neonatal CPAP use increases the potential for survival by greater than 50%, even in low-resource settings [3]. To treat newborn respiratory failure, including premature infants, noninvasive respiratory devices are often used as a standalone therapeutic applied soon after treatment with intratracheal administration of surfactant [4, 5]. Their use seeks to improve oxygenation and lung compliance while avoiding invasive mechanical ventilation via tracheal intubation and its attendant mechanical stress and strain. The goal of noninvasive respiratory support is to deliver oxygen to the infant with the least airway pressure until the lungs reach sufficient maturation for independent breathing. This support may take days, weeks, or months. Positive pressure face masks or nasal prongs are the interface for noninvasive respiratory devices to deliver continuous positive airway pressure (CPAP) and bi-level positive airway pressure (BiPAP) in the treatment of [6]. Many consider nasal CPAP (nCPAP) masks the preferred interface for CPAP delivery in preterm infant [7, 8]. Moreover, nCPAP use minimizes chronic lung disease even in very premature infants (23–24 weeks gestation) [9].

CPAP can be delivered as a water column, as a resistor (bubble CPAP), continuous flow nCPAP, non-invasive ventilation using a mechanical ventilator, variable flow nCPAP, and bilevel CPAP, or infant flow driver device [10]. Regardless of the mode of CPAP delivery all noninvasive respiratory devices interfaces require proper fitting about the nose and, or nose and mouth. Ultimately the mask interface is connected via tubing which carries pressure-regulated humidified oxygen-air mixture from the ventilator. There are several host factors, such as lung compliance, which influence the delivery of oxygen and ventilation to the infant. However, the fitting of the mask to the infant face is one of the most important factors determining effective airway pressure and oxygen delivery as well as patient tolerance to PAP. Mask fit and delivered oxygen-air mixture require continuous monitoring and adjustment to each individual patient by respiratory therapists and nurses to maintain adequate fit. This includes both adjusting the position of the mask and the supporting straps which extend around the infant’s head to hold the mask in position. In all cases the mask should create a reasonably airtight seal with the face. Poorly fitted masks result in inadequate oxygenation, aerophagia and gastrointestinal distention, and mild to severe nares and nasal septum damage [11]. In older children adhesive foam is placed on patient faces with BiPAP/CPAP to prevent mask related pressure injuries [12].

Neonates have several unique factors which make CPAP mask fit even more challenging. Moreover, as neonates have thin and fragile skin, they are at risk for skin breakdown eliciting pressure ulcers due to mask and strap surface pressure [11, 13, 14]. Infant movement increased by excess pressure or tension also impacts neonatal tolerance and perceived discomfort due to poor mask fit. In addition, neonates and infants have particularly short nasal bridges and relatively underdeveloped chins which reduce the available facial surface area for mask contact [15]. As these unique facial features change due to rapid somatic growth, they lead to mask obsolescence. Thus, as very premature infants may require CPAP masks for several months until their lungs have matured the shape and size and hence “fit” of the mask commonly evolves over the course of their hospitalization.

Recent advances in 3D printing support custom-fit neonatal CPAP devices manufactured effectively at the patient point-of-care, including austere environments [16]. Taking the above factors into consideration we sought to develop a design, process, and workflow to evaluate individualized CPAP masks for preterm infants. Though the FDA has recently approved 3D printed silicone airway stents, our workflow uses 3D silicone mold production which has many advantages, including both low cost 3D printers and classic mold and release technology [17]. A primary goal was to reduce skin mask pressure and mask strap tension while maintaining a minimal face-seal leak. We adopted the principles that 1) mask skin pressure and leak is inversely proportional to the area of surface contact and 2) excess strap pressure beyond that needed to counter CPAP pressure is used to flex or bend the CPAP mask to conform-fit facial contour. We sought to minimize both skin pressure and strap tension. We first chose the neonatal CPAP model for the experimental condition as it is considered the most challenging. The design concept was a 2-part face mask fabrication. Part 1 was an insert to be fitted to each unique facial contour produced in medical grade silicone. Part 2 was designed to interface with commercial neonatal CPAP masks. Finally, we evaluated several facial scanning technologies to determine the most readily available and utilitarian for individualized mask development.

## Materials and methods

### Structured light scanning development and testing

We used a 3D image acquisition system, workstation mask design and 3D printing technology to develop individualized CPAP masks for premature infants. After obtaining IRB approval (15–006,521) Development of CPAP Masks for Premature Infants Using 3d Printing Technology and 17–003,911 Surface Scanning Morphometry of Premature Infants parental consent was obtained to record the facial contours of fourteen infants in the level II and III NICU Mayo Clinic, Rochester, MN. To safely obtain infant facial surface topography and facial dimensions several non-invasive scanning technologies were assessed (Table 1). These included head CT scan DICOM® images obtained on an opportunistic case basis, laser scanning (Artec Space Spider, Source Graphics, Anaheim, CA, single camera (Sony, Tokyo, Japan) multi-image acquisition and a multiple camera/detector synchronous-image acquisition system (Direct Dimensions, Inc., Owings Mills, MD) using custom multi Raspberry Pi cameras for photogrammetry (triangulation of software recognized image features to construction 3D topography) [18–20]. Photogrammetry software used included ReCap™, Autodesk, San Rafael, CA, and PhotoModeler Scanner, PhotoModeler Technologies, Vancouver BC. However, infant and children facial features were too indistinct for consistent point recognition used by the photogrammetry software to extract three-dimensional measurements from two-dimensional data [21, 22]. Moreover, the clinical environment, particularly the neonatal intensive care unit, is a difficult ambient lighting environment and patient movement degraded image quality. Subsequently, we determined that structured light scanning cameras using a proprietary camera, Bellus 3D™ camera (Bellus 3D, Campbell, CA) was very suitable. The accuracy of the Bellus camera is < 0.5 mm [23]. Later, we use either the iPhone X (Apple, Cupertino, CA) using built-in TrueDepth™ infrared camera and Bellus 3D Face App, or Heges 3D Scanner (Marek Šimoník, Opava, Czech Republic), an iPhone application.

**Table 1:**
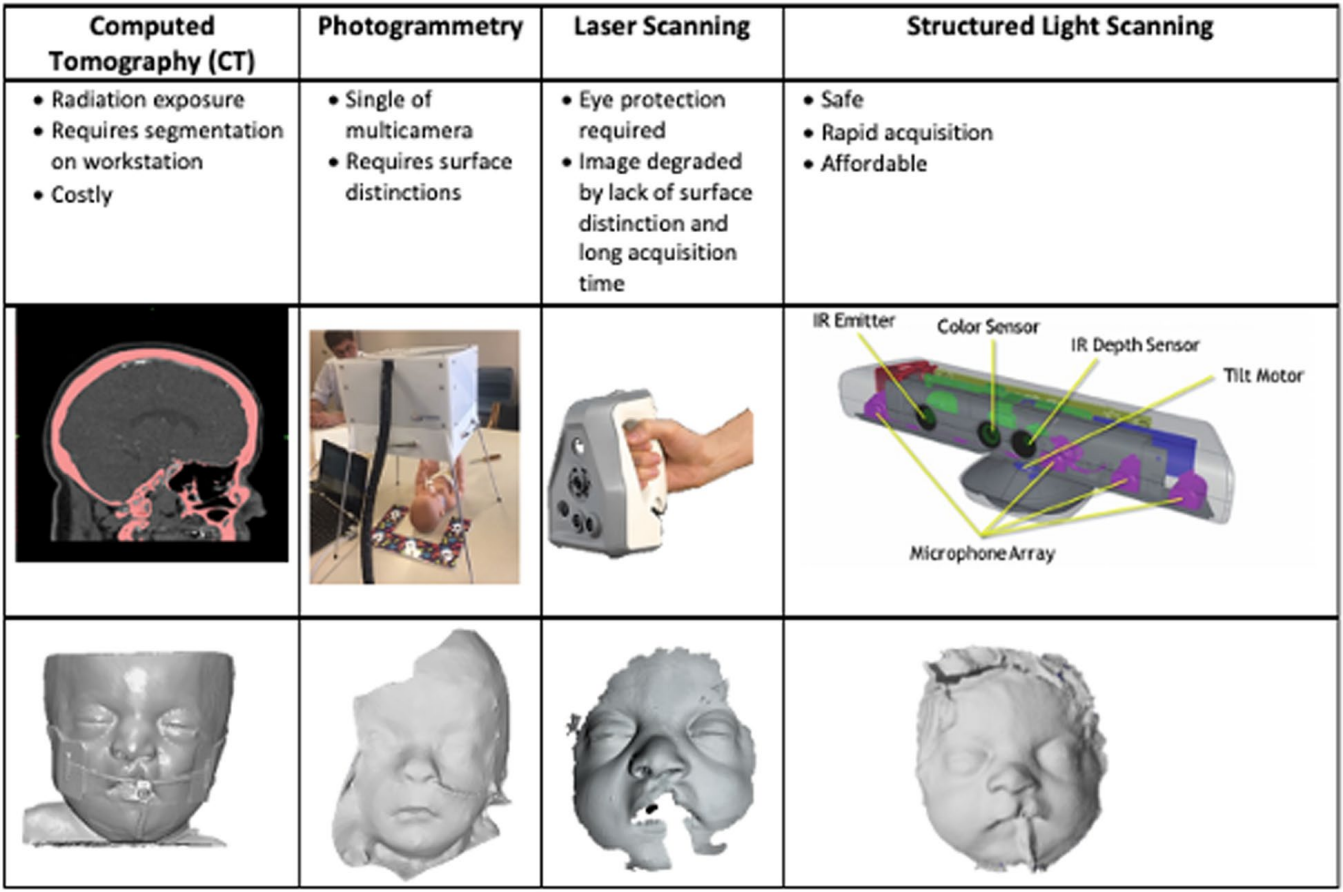
Noninvasive scanning technologies

**Fig. 1:**
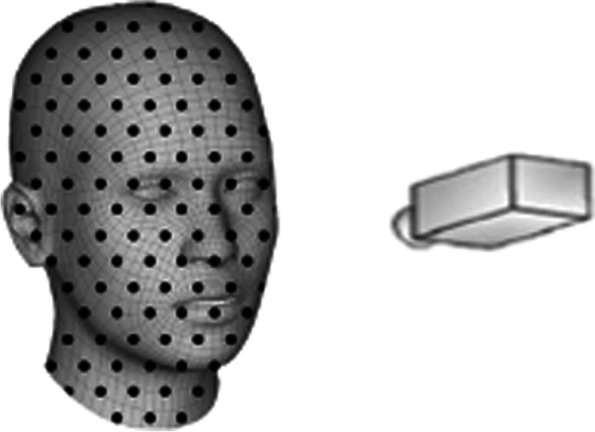
Infants face were scanned with an infrared structured light camera (Bellus 3D™) which projected 50,000 infrared dots onto the face area. Using proprietary (Bellus 3D™) landmark recognition software surface images are created with close range scanning at up tp 0.4 mm resolution

**Fig. 2:**
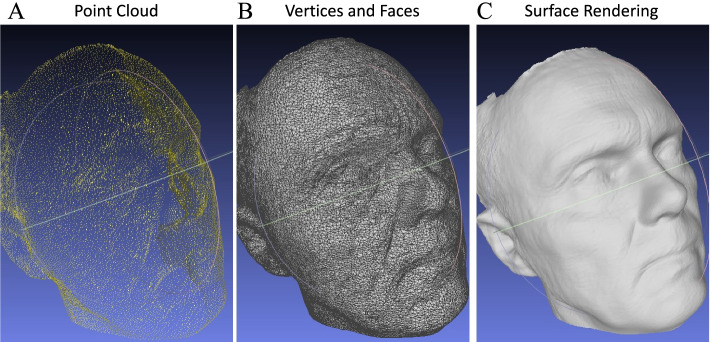
**a**) A point cloud created as 500,000 3D surface points recognized by structured infrared light scanning software. **b**) High density surface face model created with 250,00 high-definition triangles. **c**) Texture map applied to the surface of high-definition triangles (**b**) which can be used for direct 3D printing

The Bellus 3D camera (Fig. 1) is an infrared emitter and detector plus ambient light RBG camera with no risks to the infants at a working distance of 30–45 cm. The emitter projects 500,000 3D points using an infrared structured-light camera with 0.4 mm or better resolution. This typically generates 250,000 mesh triangles. Each image acquisition took 10 s and image processing took an additional ~ 20 s (Samsung Galaxy S8, Seoul, South Korea). The scanner software reformatted the imaging data (500,000 data points, Fig. 2a) and converted it into a virtual image file format (standard tessellation language, (.stl)) with 250,000 triangles with and without RGB color (Fig. 2b, c). Alternatively, the iPhone X emits 30,000 infrared dots in a known pattern which can be amalgamated during movement of the camera about the face using various acquisition software such as Heges 3D Scanner (Marek Šimoník, Opava, Czech Republic) or Bellus 3D (Bellus 3D, Campbell, CA) for the iPhone X. Both software programs perform all image processing on the iPhone X and the.stl files can be download to a CAD workstation for post-processing.

**Fig. 3:**
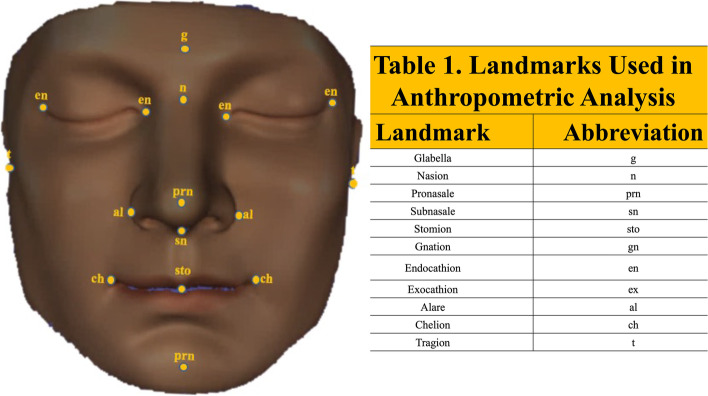
Anthropometric landmarks used on camera generated 3D face model to assess accuracy of dimensions determined by scanning

**Fig. 4:**
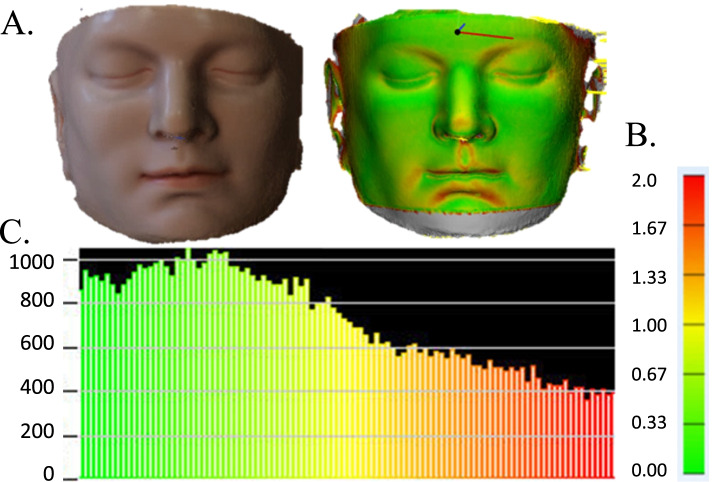
Part comparison analysis of measurements determined by structed light scanning camera (Bellus) compared to CT scanned surface map. **a**) The structured light scanning 3D data was superimposed onto the CT scanned image surface and the comparison to the 3D model was assessed by color mapping. **b**) Color scale representing the difference in mm between each point of the 3D surface with the corresponding CT surface data. Green = 0.0 mm difference. Red = 2.0 mm difference. **c**) A histogram depicting the number of points that correspond to each color

The precision and accuracy of facial scanning technologies when manual measurements are compared to imaging measurements are influenced by both machine and software characteristics [24]. Many consider CT the gold standard but the CT images require significant workstation effort to perform segmentation, 3-D reconstruction and converting to.stl formant. To determine the accuracy of the Bellus 3D™ camera facial data we used 21 standard anthropometric measurements (Fig. 3) obtained on a life-size model of the human head including 11 standard landmarks (Fig. 3) [25, 26]. Measurements were made 3 times with digital calipers and the mean compared to image data obtained and processed 3 times with the Bellus 3D™ camera and measured in Materialise Mimics (Materialise, Leuven Belgium). Statistical comparison was made between digital caliper data and the 3D measurements (Table 2). The measurements were visually confirmed with a Part Comparison Analysis performed on Materialise 3-Matic (Materialise, Leuven, Belgium) against a computed tomography (CT) scan of the same model (Fig. 4).

**Table 2:**
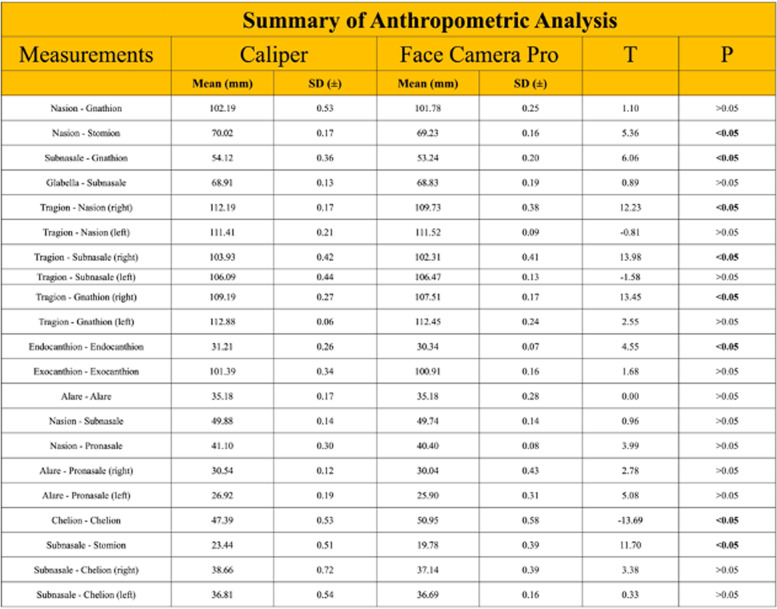
Summary of anthropometric analysis

### Face models to test CPAP inserts

**Fig. 5:**
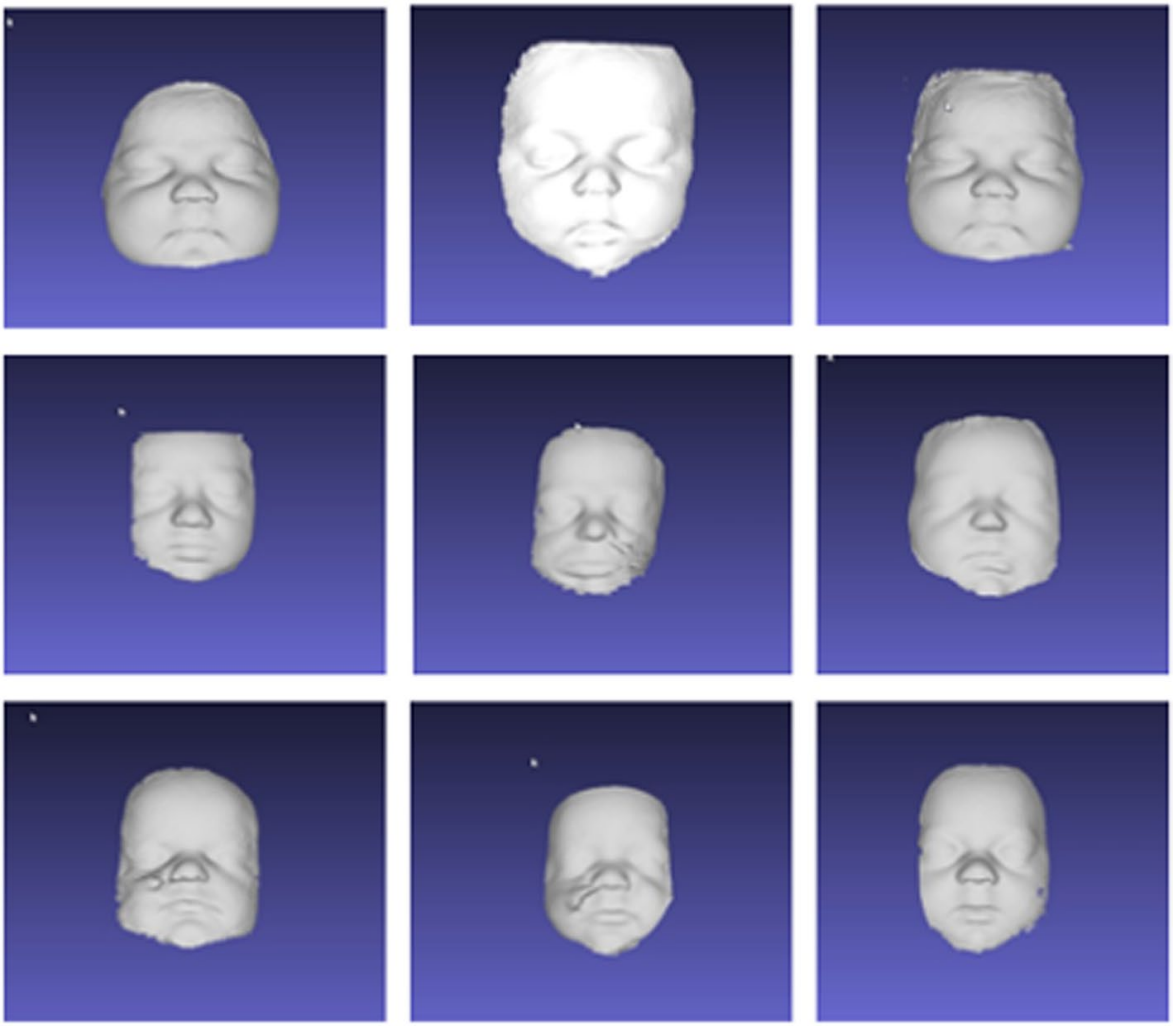
9 representative structured facial scan of neonates using Bellus 3D camera and Bellus 3D scanning software

To create custom face masks the structured light scanned.stl face surface images obtained with either the Bellus platform or the iPhone Heges software platform were converted to solid face bodies using image software (MeshLab, Open source), and Meshmixer™ (Autodesk, Inc., San Rafael, CA). Neonatal full-face models (Fig. 5) were 3D printed using a PolyJet™ photopolymer soft material (Aguilis30, ™ Stratsys, Eden Prairie, MN) on a Stratsys Connex Objet 350 printer with a Shore value of 40. A 3D printed nasopharynx was attached to each face which had been created using an anonymized CT from aged-matched subjects. The nasopharyngeal component of the infant mask was attached to an artificial lung prosthesis to mimic normal lung inflation and expiration pressures using a Hamilton T1 ventilator, (Hamilton Medical, Bonaduz, Switzerland).

### CPAP mask fabrication and testing

**Fig. 6:**
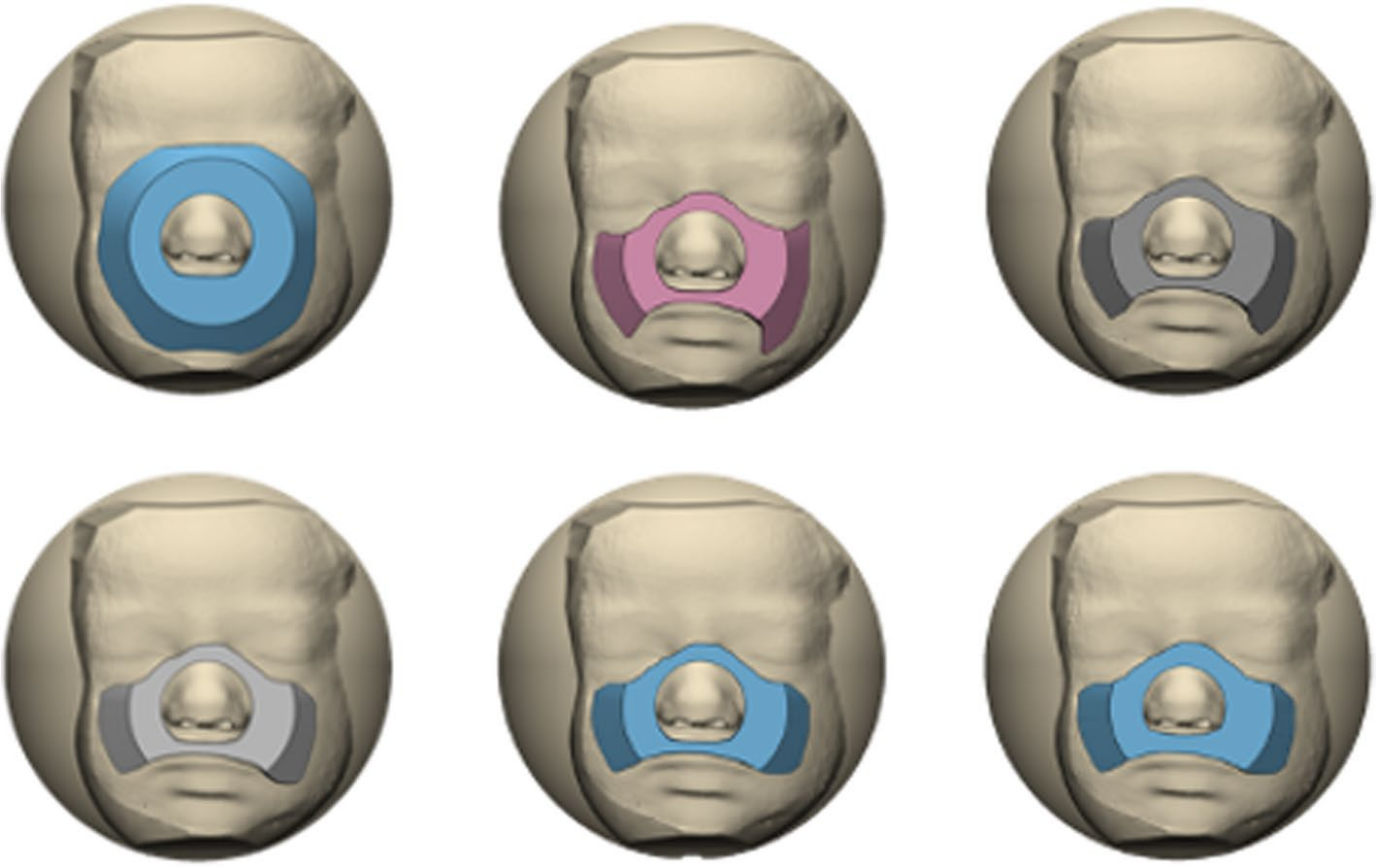
Using structured light scans of neonatal faces individual CPAP inserts were designed to uniquely conform, to facial topography

**Fig. 7:**
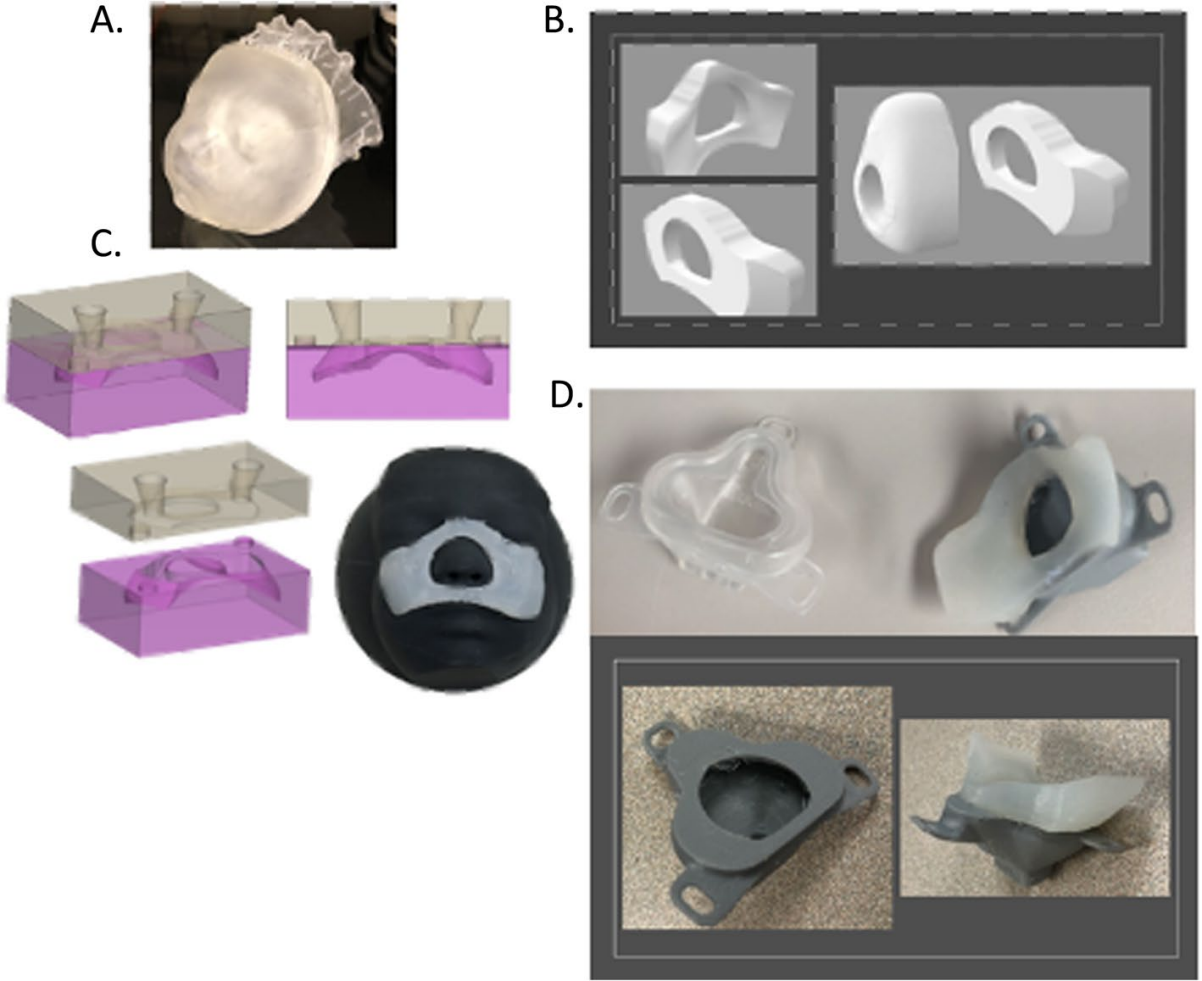
A. Preterm 3D printed neonatal face. Digitally the solid face bodies were use as a digital “cutting tool” to contour the reaward potion of the CPAP insert. **B**, “Concept design” of nCPAP mask design with 2 parts forward part to mate proprietary nCPAP connection and rear part to conform to the surface topography of each face while providing flat mating surface to forward part. **C.** Silicon molding process **D**. Proprietary neonatal CPAP mask (Hamilton infant XL) (left),2- part molded CPAP mask (risk and below)

**Fig. 8:**
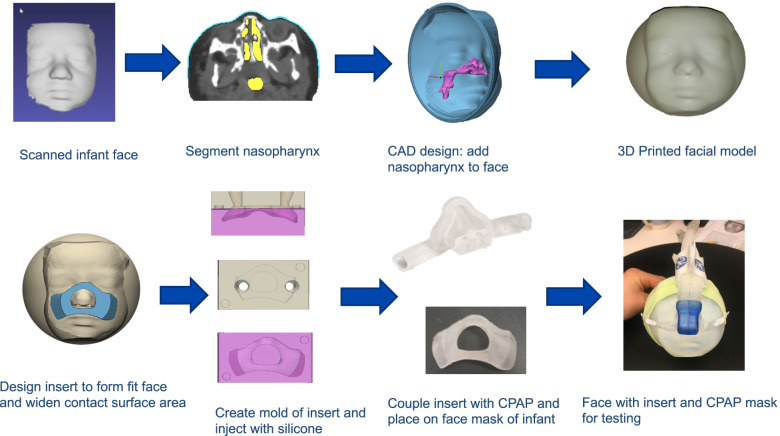
3D infant face model and CPAP insert design and fabrication workflow

3D CAD design software, Fusion 360 (Autodesk, San Rafael, CA), or Materialise 3-matic, (Materialise, Leuve**n**, Belgium), was used to develop CPAP inserts for commercially available neonatal CPAP masks. The rearward insert part was designed to match the facial contour. The rearward part was designed to uniquely conform to facial topography and present a flat surface to mate with the proprietary CPAP mask (Fig. 6). For each case the CAD operator created an individualized flange surface about the rearward insert to cover the mouth and nose and avoid the region about the eyes. Specifically, the solid face bodies (Fig. 7A) were used as the digital “cutting tools” to contour the rearward portion of the insert to match the facial contour (Fig. 7B). This resulted in a personalized design matching the neonate’s facial contour with the CPAP insert (Fig. 5c). The inserts were designed to have more surface area than conventional CPAP masks for surface pressure to be dissipated. The mask design was converted into an.stl file which was subsequently subtracted from a box design to create a mold (Fig. 6). The insert was printed using polylactic acid (PLA, Hatchbox3D.com) at 0.16 mm layer height on Prusa i3 MKS3, (Prusa Research, Prague, Czech Republic) or Creality SR10S Pro (Shenzhen, China) using slicing software (Ultimaker Cura, Ultrecht, Netherlands). Subsequently, the insert was cast in a negative silicone mold (Mold Star™ 20 T, Smooth-On, Lower Macungie, PA). Then a positive was cast using silicones (Ecoflex™, Smooth-On, Lower Macungie, PA) of different stiffness grades (Shore values) (Fig. 7C). The insert molded part was joined to the proprietary mask with cyanoacrylate adhesive (Fig. 7D). Total workflow time from structured light scanning to finished product was typically less than 6 h (Fig. 8).

### Model testing

**Fig. 9:**
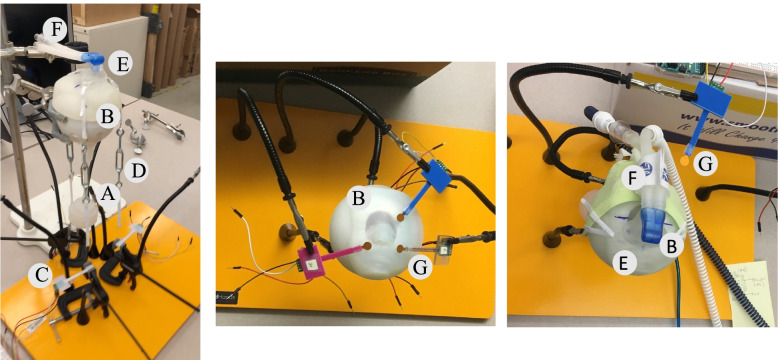
Test platform for determining surface pressure between neonatal CPAP mask and model neonatal face and determining minimal strap tension to elicit air leak less than 0.5 L/min. **A** artificial lung prosthesis. **B** 3D printed model neonatal face. **C** Force transducer to measure strap tension. **D** Adjustable nasal CPAP strap. **E** Proprietary neonatal CPAP mask. **F** ventilator tubing. **G** Pressure transducer to measure surface pressure between neonatal CPAP mask and model neonatal face

CPAP masks were place on the neonatal full-face models with and without individualized silicone molded CPAP inserts. The CPAP proprietary masks (infant = the Hamilton CPAP XL, Hamilton Medical Inc, USA, Reno, NV) were attached to the Hamilton T1 ventilator and CPAP pressures of 4, 6, 8 and 10 cm H_2_O were delivered. Four calibrated 8 mm diameter pressure sensors (SingleTact®, PPS UK Limited, Glasgow, United Kingdom) were placed at 4, 8 and 12 O’clock positions between the CPAP mask with or without the 3D customized CPAP insert and the neonatal face model. The CPAP mask straps were adjusted to maintain an expiratory circuit leak of ≤ 0.5 L/min. Pressure from the sensors were calibrated in pounds per square inch (PSI). Simultaneously, co-linear tension sensors (SingleTact®) were placed for each strap holding the mask in position around the infant head and force measurements were determined at 4, 8 and 12 O’clock position at each CPAP pressure. Each sensors output was calibrated daily, and digital outputs were recorded using an Arduino microcontroller (www.arduino.cc) (Fig. 9).

## Results

### Neonatal facial topography imaging technologies

We tested several imaging technologies to obtain neonatal facial topography. Though we had access to head CT imaging on an opportunistic basis we discounted this due to radiation exposure and need for repeated imaging as growth occurs. Both laser scanning and single camera photogrammetry are unsuitable due to long acquisition times and image degradation due to movement. Multicamera photogrammetry improves acquisition time, but this still demands software recognition of facial distinctions which are absent due to the flat skin complexion of newborns. Also, many photogrammetry platforms utilize cloud-based analysis which poses patient confidentiality concerns. Both the Bellus 3D™ camera or the iPhone structured light camera using Bellus 3D application software have short acquisition times (< 10 s) and high point resolution (≤ 0.5 mm). However, the Bellus software requires facial landmark registration to limit image capture of only the human face in the field of view. As such, it is designed to register the larger facies of children and adults and was only able to capture neonatal facial images in less than 50% of cases. The best imaging platform was the iPhone running Heges application software. Image acquisition time was short (< 10 s) and the platform does not require facial landmark registration.

### Precision and accuracy of structure light scanning of facial landmarks

We determined the accuracy of the facial contour of the human head model obtained with the structured light scanning (Bellus 3D Camera) when compared with the contour obtained by computed tomography (CT) of a facial model to be highly accurate (< 1 mm, Fig. 4). These results are equivalent to the Heges iPhone application software published accuracy of 0.5 -0.8 mm. Our results are consistent with others using the same and similar platforms [19, 27].

### Skin pressure and strap tension with and without 3D CPAP insert

**Fig. 10:**
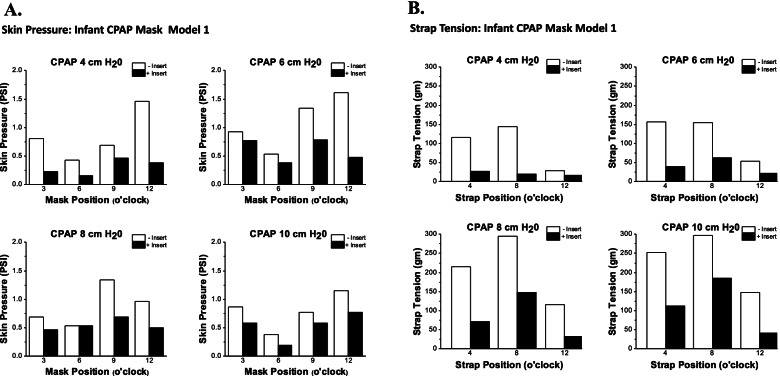
**A** Infant Model 1. Representative case showing that CPAP mask surface pressures were lesser at each mask position and at each CPAP setting. **B** Infant Model 1. Representative case showing that CPAP mask strap tensions were lower at each strap position at each CPAP setting

Three infant faces were printed with Polyjet™ photopolymer soft material on the Stratsys Connex Objet 350 printer with a Shore value between 30 and 40. The nasopharyngeal component was attached to an artificial lung prosthesis. The CPAP masks with and without the 3D inserts were attached to the face using proprietary straps and the CPAP mask was connected to the ventilator and PEEP was adjusted. Strap pressure for each mask (example Fig. 10a, b) was adjusted to seek less than 0.5 L/min air leak. In each of the 3 infant face models surface pressures measured between the mask, or mask insert, and facial surface were significantly lower at all sites (9.6%—62%, Table 3) when the face models were tested with CPAP inserts compared to face models tested without CPAP inserts. Similarly, mask strap pressure to achieve target air leak at each level of PEEP was significantly lower (16.0% – 56.6%, Table 3) at all positions when the face models were tested with CPAP inserts compared to face models tested without CPAP inserts.

**Table 3:**
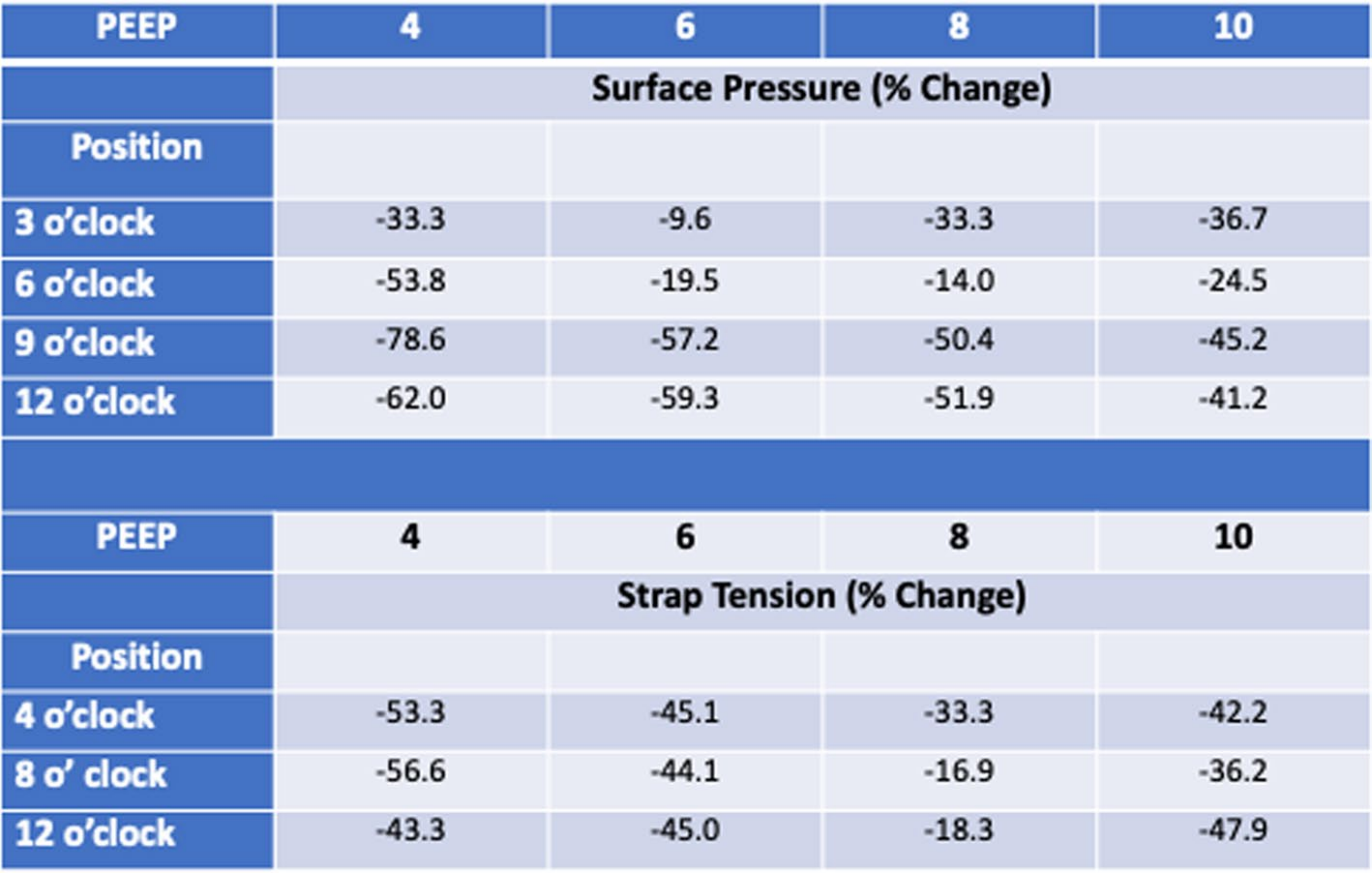
Percent change in variable (surface pressure, strap tension) with insert compared to without insert

## Discussion

We developed a process to design and evaluate individualized custom-CPAP masks for premature infants. Though others have used the iPhone camera to acquire multiple digital photographs to process using photogrammetry software to create 3D biologic models[28] our study adds to the emerging applications describing a framework using iPhone TrueDepth image acquisition that is straightforward and uses readily obtainable software. The image acquisition system using a structured infrared light facial scanning camera (iPhone) was mobile, non-invasive, rapid, safe, and highly accurate. Our project sought to determine both optimal acquisition device (camera) and software (camera acquisition and image processing). Our acquisition work initially tilized the Bellus3D Face Camera and later the Bellus 3D FaceApp for the iPhone. Both are no longer available as of March 31, 2022. However, we have shown that currently publicly available image acquisition iPhone application software, such as Heges, have similar performance. Our workflow and the use of the iPhone TrueDepth camera extend the recent work by Alhazmi et al. to create thermoplastic polyurethane facemasks for patients with facial burns[29]. In our study image acquisition applications for the iPhone allow an individualized custom insert to be created using a 3D printed mold to produce and an insert which can be placed between the commercial CPAP mask and the infant face. This custom insert reduced the surface pressure and strap tension at clinically relevant CPAP settings and ventilator circuit leak. Specifically, individualized CPAP mask inserts manufactured using 3D printed molds and silicon injection were effective at decreasing surface pressure and mask strap pressure (ranging 10 to 78%) in the models studied compared to CPAP masks without inserts.

### Limitations

Our project was a proof-of-concept workflow demonstration to determine the utility of neonatal facial structured light scanning in fabrication of infant CPAP mask development. The FDA classifies positive airway devices including CPAP as a Class II medical device. As such the patient-contacting components of the device (mask) must be demonstrated to be biocompatible, demonstrate adequate device performance over the labeled use life, demonstrate the device can withstand typical expected forces during use withstand worst-case scenario air pressures, have reprocessing instructions for reusable components and have labeled cleaning instructions. We did not demonstrate any of the above in our workflow analysis. Moreover, for clinical applications strict criteria for CPAP mask design would be necessary, including standardizing shape requirements as well as safe surface coverage. Future work includes creating more types of CPAP insert designs, testing more types of silicone, and modifying the type and location of the straps.

## Conclusion

We found that readily available structured light scanning devices such as the iPhone X are a low cost, safe, rapid, and accurate tool to develop accurate models of preterm infant facial topography. In this preliminary study structured light scanning developed 3D nCPAP inserts applied to commercially available CPAP masks significantly reduced skin pressure and strap tension at clinically relevant CPAP pressures when utilized on model neonatal faces. This workflow maybe useful at producing individualized nCPAP masks for neonates reducing complications due to misfit.

## Data Availability

All raw data and analysis are available upon request from wylam.mark@mayo.edu.
